# FUTURE OF THE LANGUAGE MODELS IN HEALTHCARE: THE ROLE OF CHATGPT

**DOI:** 10.1590/0102-672020230002e1727

**Published:** 2023-05-08

**Authors:** Francisco Tustumi, Nelson Adami Andreollo, José Eduardo de Aguilar-Nascimento

**Affiliations:** 1 Universidade de São Paulo, Faculty of Medicine, Department of Gastroenterology – São Paulo (SP), Brazil;; 2 Universidade Estadual de Campinas, Faculty of Medical Sciences, Department of Surgery, Digestive Disease Surgical Unit – Campinas (SP), Brazil;; 3 Universidade Federal de Mato Grosso, Medical School, Department of Surgery – Cuiabá (MT), Brazil.

**Keywords:** Guidelines as topic, Artificial intelligence, Diagnosis, Costs and cost analysis, Delivery of health care, Guias como assunto, Inteligência artificial, Diagnóstico, Custos e análise de custos, Atenção à saúde

## Abstract

The field of medicine has always been at the forefront of technological innovation, constantly seeking new strategies to diagnose, treat, and prevent diseases. Guidelines for clinical practice to orientate medical teams regarding diagnosis, treatment, and prevention measures have increased over the years. The purpose is to gather the most medical knowledge to construct an orientation for practice. Evidence-based guidelines follow several main characteristics of a systematic review, including systematic and unbiased search, selection, and extraction of the source of evidence. In recent years, the rapid advancement of artificial intelligence has provided clinicians and patients with access to personalized, data-driven insights, support and new opportunities for healthcare professionals to improve patient outcomes, increase efficiency, and reduce costs. One of the most exciting developments in Artificial Intelligence has been the emergence of chatbots. A chatbot is a computer program used to simulate conversations with human users. Recently, OpenAI, a research organization focused on machine learning, developed ChatGPT, a large language model that generates human-like text. ChatGPT uses a type of AI known as a deep learning model. ChatGPT can quickly search and select pieces of evidence through numerous databases to provide answers to complex questions, reducing the time and effort required to research a particular topic manually. Consequently, language models can accelerate the creation of clinical practice guidelines. While there is no doubt that ChatGPT has the potential to revolutionize the way healthcare is delivered, it is essential to note that it should not be used as a substitute for human healthcare professionals. Instead, ChatGPT should be considered a tool that can be used to augment and support the work of healthcare professionals, helping them to provide better care to their patients.

## RESUMO

A área da medicina sempre esteve na vanguarda da inovação tecnológica, buscando constantemente novas estratégias para diagnosticar, tratar e prevenir doenças. As diretrizes para a prática clínica são para orientar as equipes médicas quanto ao diagnóstico, tratamento e medidas de prevenção aumentaram ao longo dos anos. O objetivo é reunir o máximo de conhecimento médico para construir uma orientação para a prática. As diretrizes baseadas em evidências seguem várias das principais características de uma revisão sistemática, incluindo busca sistemática e imparcial, seleção e extração da fonte de evidência. Nos últimos anos, o rápido avanço da inteligência artificial forneceu aos médicos e pacientes acesso a informações personalizadas e baseadas em dados, suporte e novas oportunidades para os profissionais de saúde melhorarem os resultados dos pacientes, aumentarem a eficiência e reduzirem custos. Um dos desenvolvimentos mais empolgantes da Inteligência Artificial foi o surgimento dos *chatbots*. Um *chatbot* é um programa de computador para simular conversas com usuários humanos. Recentemente, a OpenAI, uma organização de pesquisa focada em aprendizado de máquina, desenvolveu o ChatGPT, um grande modelo de linguagem que gera texto semelhante ao humano. O ChatGPT usa um tipo de inteligência artificial conhecido como modelo de aprendizado profundo. O ChatGPT pode pesquisar e selecionar rapidamente evidências em vários bancos de dados para fornecer respostas a perguntas complexas, reduzindo o tempo e o esforço necessários para pesquisar um tópico específico manualmente. Consequentemente, os modelos de linguagem podem acelerar a criação de diretrizes de prática clínica. Embora não haja dúvida de que o ChatGPT tem potencial para revolucionar a forma como os cuidados de saúde são prestados, é essencial observar que não deve ser usado como substituto de profissionais de saúde humanos. Em vez disso, o ChatGPT deve ser visto como uma ferramenta que pode ser usada para aumentar e apoiar o trabalho dos profissionais de saúde, ajudando-os a prestar melhores cuidados aos seus pacientes.

## INTRODUCTION

The field of medicine has always been at the forefront of technological innovation, constantly seeking new strategies to diagnose, treat, and prevent diseases. The number of publications in the main medical databases, such as PubMed and Embase, has been increasing steadily over the years due to the contributions from authors worldwide, the expansion of research areas, the rise of open-access publishing, and advancements in technology^2^. This growth in biomedical literature provides a wealth of information that can be used to advance research and improve patient care^7^. However, this fast science production in the medical field has boosted a new problem: how can clinical practice follow the constantly updated improvement of science?

**Figure 1:**
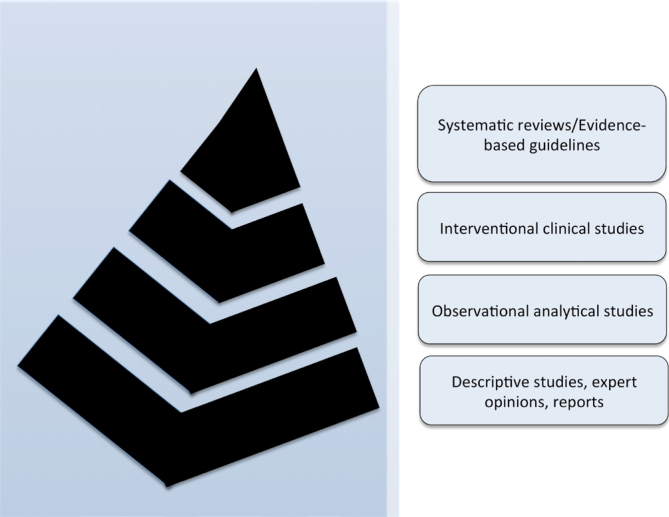
Pyramid of the level of evidence. Systematic reviews and evidence-based guidelines are usually considered the top evidence, gathering all the current evidence for clinical practice^14^.

**Figure 2:**
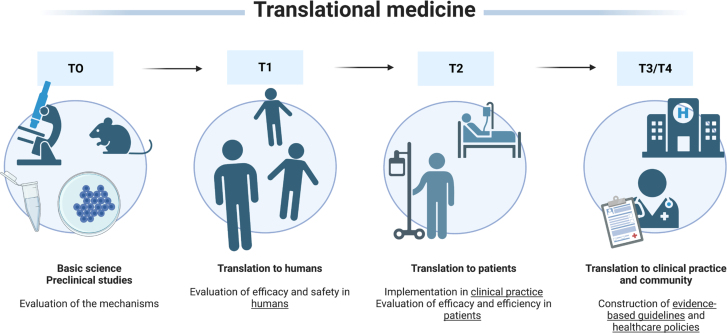
Translational medicine stages. Evidence-based guidelines and healthcare policies are the endpoints of any research line. These guidelines and policies orientate healthcare professionals for patient management^18^.

Guidelines for clinical practice to orientate medical teams regarding diagnosis, treatment, and prevention measures have increased over the years^6^. The purpose is to gather the most medical knowledge to construct an orientation for practice. Guidelines help standardize care, improve patient outcomes, promote efficient use of resources, and reduce the risk of adverse events. In this sense, evidence-based reviews and guidelines are located at the top of the pyramid in the level of evidence (Figure 1)^14^ and in the last stages of translational medicine (Figure 2)^18^.

Evidence-based guidelines follow several main characteristics of a systematic review, including systematic and unbiased search, selection, and extraction of the source of evidence^21,24^. However, there are numerous obstacles to constructing evidence-based guidelines. Search, selection, and extraction take many efforts and a long time^5^. Consequently, evidence-based guidelines are usually published with a long-time delay. An evidenced-based guideline can take anywhere from several months to more than a year to complete, depending on the scope and complexity of the review. Conducting a comprehensive search for relevant studies can take several months, depending on the literature database's size and the search terms’ complexity. Screening studies for inclusion based on pre-specified criteria can take another couple of months, depending on the number of studies identified and the number of reviewers involved. Updates of these guidelines may take years or even never be performed, and healthcare professionals may take their diagnosis, treatment, and prevention measures based on guidelines out of step with the current technological and scientific level.

Another relevant issue in evidence-based guidelines and systematic reviews refers to the risk of selection bias^10,22,23^. Some authors may make a poor selection of the pieces of evidence for supporting guidelines for some reasons which may be due to quite restricted eligibility criteria, such as period, language, or databases searched, or even due to human failure during the process of search, selection, and extraction of the source of evidence^17^. Besides, poor selection can also be due to improper manipulation of outcomes, influenced by personal beliefs or opinions^8^.

In this context, in recent years, the rapid advancement of artificial intelligence (AI) has provided new opportunities for healthcare professionals to improve patient outcomes, increase efficiency, and reduce costs^25^. In the medical field, AI has the potential to provide clinicians and patients with access to personalized, data-driven insights and support.

One of the most exciting developments in AI has been the emergence of chatbots, which have the potential to revolutionize the way healthcare is delivered^1^. A chatbot is a computer program used to simulate conversations with human users. Recently, OpenAI, a research organization focused on machine learning, developed ChatGPT, a large language model that generates human-like text. The initial version of Generative Pre-Trained Transformer (GPT) was first introduced in 2018. Since then, several improved versions of GPT have been released. ChatGPT is a variant of the GPT models that have been specifically fine-tuned and optimized for chat-based applications^13^.

**Figure 3:**
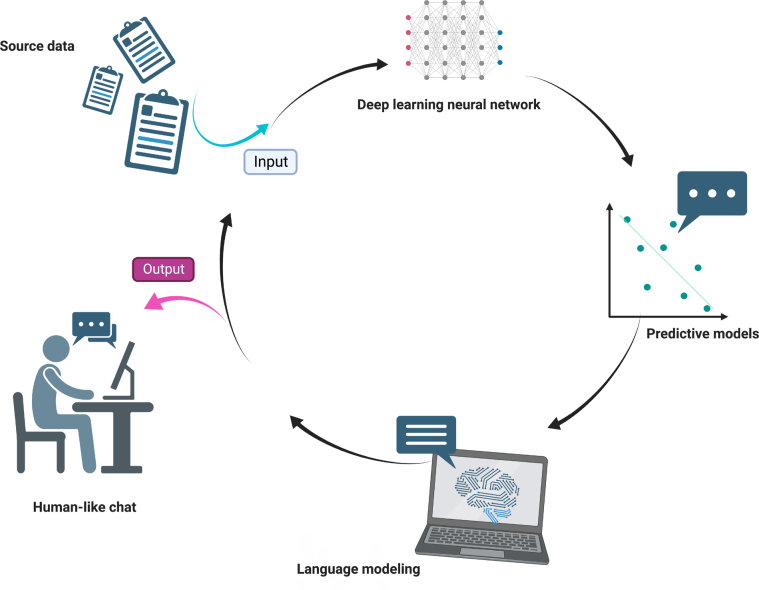
Modern language models use artificial intelligence to create a human-like text.

ChatGPT uses a type of AI known as a deep learning model (Figure 3). Specifically, it is a type of transformer-based language model that uses a neural network architecture known as the Transformer. This architecture was first introduced in 2017 and has since become a popular choice for natural language processing tasks, such as language translation, language generation, and text classification. The Transformer architecture uses multiple layers of neurons, or “transformer blocks,” that allow it to process and extract features from input data hierarchically. This architecture has proven to be very effective for natural language processing tasks, and it forms the basis of many of the most advanced language models, including ChatGPT. ChatGPT is designed to understand and respond to text-based inputs from users in order to provide helpful and informative responses to their questions and comments^4,11,12^.

There are different categories of chatbots, and a chatbot can belong to more than one category: Knowledge Domain (generic, open domain, and closed domain); Service Provided (interpersonal, intrapersonal, and inter-agent); Goals (informative, chat-based/conversational, and task-based); Response Generation Method (rule based, retrieval based, and generative); Human-Aid (human-mediated and autonomous); Permissions (open-source and commercial); and Communication Channel (text, voice, and image)^1^. Therefore, there are many applications of chatbots, like education environments, customer service, medicine and health, robotics, industrial, and others^1^.

The chatbots, AI, and telemedicine are increasingly being used in healthcare services with good acceptance, such as education, diagnostic imaging and genetic diagnosis, as well as clinical laboratory, screening, and health communications^9,15,25^.

One of the key benefits of using ChatGPT in medicine is the ability to provide fast, accurate, and up-to-date information to healthcare professionals and patients. ChatGPT can quickly search and select pieces of evidence through numerous databases to provide answers to complex questions, reducing the time and effort required to research a particular topic manually. Consequently, language models can accelerate the creation of clinical practice guidelines. AI may help screen numerous databases quickly, saving time and accelerating the finishing of the guidelines^11,20^.

In addition to providing information, ChatGPT can also assist healthcare professionals with making diagnoses by analyzing symptoms and recommending tests or treatments. This can help to improve the accuracy of diagnoses and reduce the number of misdiagnoses. ChatGPT can also be used to help manage patients with chronic conditions by providing information about medications, lifestyle changes, and treatment options^3,15^.

Besides, the use of AI might reduce the risk of selection bias. The dataset used to train ChatGPT is typically chosen to be as inclusive as possible, including a broad range of sources and perspectives. ChatGPT is not influenced by individual cognitive biases, which can affect human decision-making. As an AI, ChatGPT uses a purely algorithmic approach to generate responses without being influenced by personal beliefs or opinions. This can reduce the risk of selection bias, as the responses are based solely on the data used to train the algorithm^16^.

However, ChatGPT is trained on large amounts of data, which may contain biases or inaccuracies that the system could inadvertently propagate. This information bias could result in inaccurate or discriminatory recommendations or treatment options^15,16^. ChatGPT is trained based on web data, and several web information sources may be easily wrong. Besides, ChatGPT may not always have a full understanding of the context of the data in which a question or prompt is being asked. This could lead to inaccurate or inappropriate responses, particularly in complex medical situations or situations comprehending patients’ preferences or feelings.

For evidence-based guidelines with quantitative synthesis (meta-analysis), despite AI potentially detecting statistical heterogeneity, clinical heterogeneity depends on the critical analysis of the included articles^19^. Without the human critical analysis capacity, language models may gather information in a review based on articles whose data cannot be pooled due to their methodological differences!

**Figure 4:**
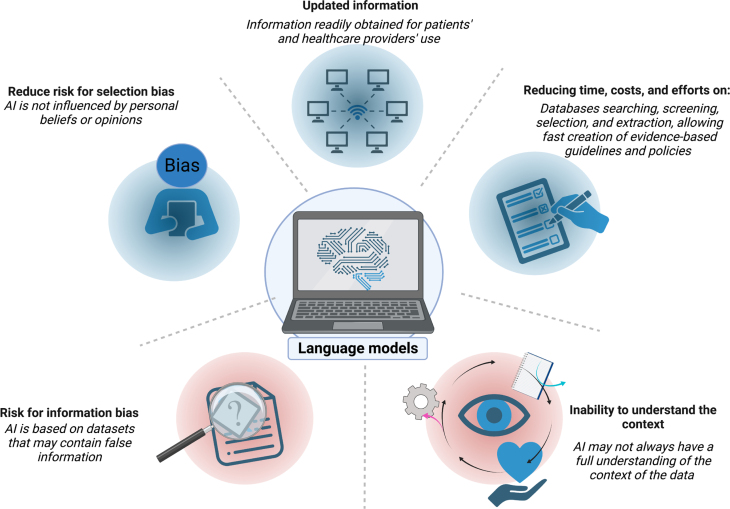
Language models in medicine are promising and have the potential to significantly improve patient outcomes and increase the efficiency of healthcare delivery. However, it is crucial to consider the limitations of this technology carefully. AI: Artificial intelligence.

While there is no doubt that ChatGPT has the potential to revolutionize the way healthcare is delivered, it is essential to note that it should not be used as a substitute for human healthcare professionals. Instead, ChatGPT should be considered a tool that can be used to augment and support the work of healthcare professionals, helping them to provide better care to their patients (Figure 4).

## CONCLUSION

The use of ChatGPT in medicine is a promising development that has the potential to significantly improve patient outcomes and increase the efficiency of healthcare delivery. However, it is important to consider this technology's limitations carefully and ensure that it is used responsibly and in conjunction with human healthcare professionals.
